# Supplementary material for: High-entropy superparaelectrics with locally diverse ferroic distortion for high-capacitive energy storage
Source: Nat Commun. 2024 Aug 8;15:6754. doi: 10.1038/s41467-024-51058-6 (PMC11310198; doi:10.1038/s41467-024-51058-6)
Supplement: Supplementary file 1 — Supplementary Information [file 41467_2024_51058_MOESM1_ESM.pdf]

Supplementary Information

High-entropy superparaelectrics with locally diverse ferroic distortion for high-capacitive energy storage

Jianhong Duan[1], Kun Wei[1], Qianbiao Du[1], Linzhao Ma[1], Huifen Yu[2], He Qi[2]*, Yangchun Tan[3], Gaokuo Zhong[3]* & Hao Li[1]*

[1]College of Electrical and Information Engineering, Hunan University, Changsha 410082, China

[2]Beijing Advanced Innovation Center for Materials Genome Engineering Department of Physical Chemistry, University of Science and Technology Beijing, Beijing 100083, China

[3]Shenzhen Institute of Advanced Technology, Chinese Academy of Sciences, Shenzhen 518055, China

*Corresponding author, E-mail: qiheustb@ustb.edu.cn (He Qi); gk.zhong@siat.ac.cn (Gaokuo Zhong); hli@hnu.edu.cn (Hao Li)

Methods

Finite element simulation

The electric field, electric potential distribution, and electric tree evolution of SLTT-0 and SLTT-0.30 ceramics were simulated by the finite element simulation method with 2D models using COMSOL software. The simulation model and parameters were based on the yellow boxed area of the SEM image shown in Supplementary Fig. 16. A scalar field $s(x,t)$ is used to represent the breakdown state (where $s = 1$ for the initial state and $s = 0$ for the complete breakdown state; $0 \leq s \leq 1$). As the dielectric constant (ε_r) is a continuous function of s, the breakdown state can be described by the difference of ε_r, which can be expressed by the following equation[1,2]:

$$\varepsilon(s) = \frac{\varepsilon_{ini}}{f(s) + \psi} \qquad (1)$$

where εini represents the initial ε_r, $f(s) = 4s^3 - 3s^4$, and ψ is 0.0001. Furthermore, the ε_r of grains is electric field-dependent in ceramics, following Johnson's approximation[3], whereas the ε_r of grain boundaries is linear. Consequently, the ε_r for grains (ε_g) and grain boundaries (ε_{gb}) at a given electric field is described by the following equation:

$$\varepsilon_{ini}(E) = \frac{\varepsilon_g(0)}{(1 + kE^2)^{1/3}} \qquad (2)$$

$$\varepsilon_{ini}(E) = \varepsilon_{gb} \qquad (3)$$

where k is 0.0013, and ε_g (0) is the zero-field dielectric constant and is taken to be 6048 and 1316 for SLTT-0 and SLTT-0.30 ceramics, respectively. In this simulation, the ratio of ε_g to ε_{gb} is set to be 10:1[4,5].The theoretical model can be established by the following equation:

$$\overline{\nabla}\left[\frac{1}{f(s) + \psi}\overline{\nabla\phi}\right] = 0 \qquad (4)$$

$$\frac{\partial s}{\partial t} = -\frac{f'(s)}{2[f(s) + \psi]^2}\overline{\nabla\phi} \times \overline{\nabla\phi} + f'(s) + \frac{1}{2}\overline{\nabla}^2 s \qquad (5)$$

Under the correct boundary and initial conditions, Equations (4) and (5) can be applied to the solution of dimensionless unknown fields $\phi(x,t)$ and $s(x,t)$. The simulation results are shown in Supplementary Fig. 10.

Supplementary Table 1 S_{config} of the SLTT-*x* ceramics.

x	0	0.20	0.25	0.30	0.35
S_{config}	0.88R	1.47R	1.54R	1.61R	1.66R

S_{config} can be calculated using the following equation[6,7]:

$$S_{config} = -R((\sum_{i=1}^{N} x_i ln x_i) + (\sum_{j=1}^{M} x_j ln x_i)) \qquad (6)$$

where R, N (M) and x_{i} (x_{j}) are the ideal gas constant, atomic species and contents at the equivalent cation (anion) sites, respectively. Note that the x in the SLTT-x system represents the mass ratio. Therefore, x needs to be converted to a molar ratio before it can be used to calculate S_{config}. It is defined as medium entropy when $1.0R \leq S_{\mathrm{config}} < 1.5R$ and high entropy when $S_{\mathrm{config}} \geq 1.5R$.

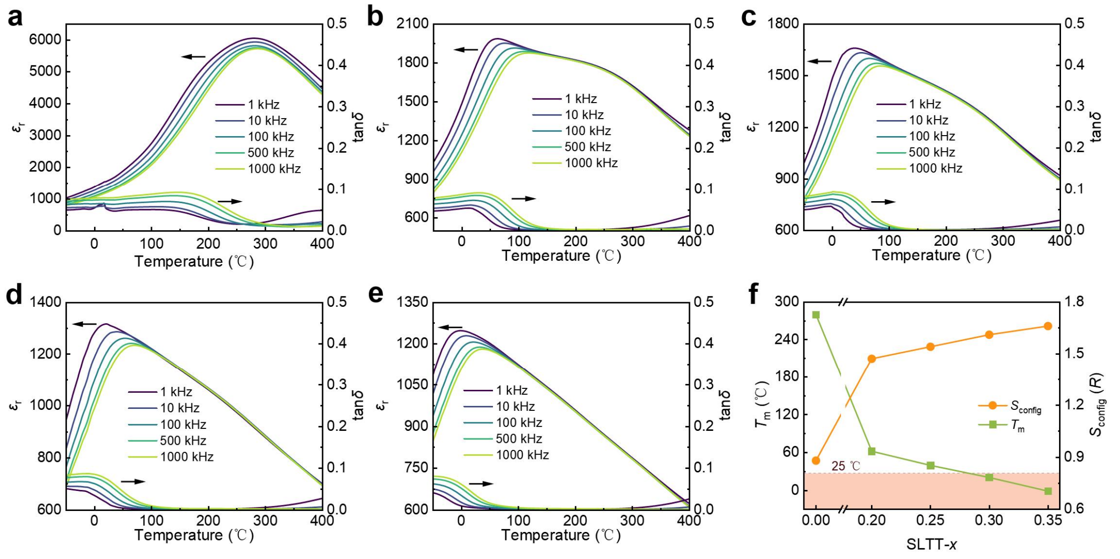

Supplementary Fig. 1 Dielectric Properties of SLTT-*x* Ceramic. Temperature-dependence of the dielectric constant (ε_r) and loss (tanδ) at different frequencies for **a** $x = 0$, **b** $x = 0.20$, **c** $x = 0.25$, **d** $x = 0.30$, and **e** $x = 0.35$. **f** S_{config} and T_m as a function of x. With an increase in S_{config}, the relaxor features are visibly enhanced, manifested by the broadened dielectric peak and the pronounced frequency dispersion[8,9]. Importantly, T_m decreases significantly and is inversely related to S_{config}. As S_{config} increases from 0.88R ($x = 0$) to 1.61R ($x = 0.30$), T_m decreases correspondingly from 279.9 °C to 20.3 °C. Further increasing S_{config} to 1.66R ($x = 0.35$), T_m drops to −1.3 °C. This phenomenon confirms the achievement of the room temperature SPE state in SLTT-0.30 and SLTT-0.35 ceramics.

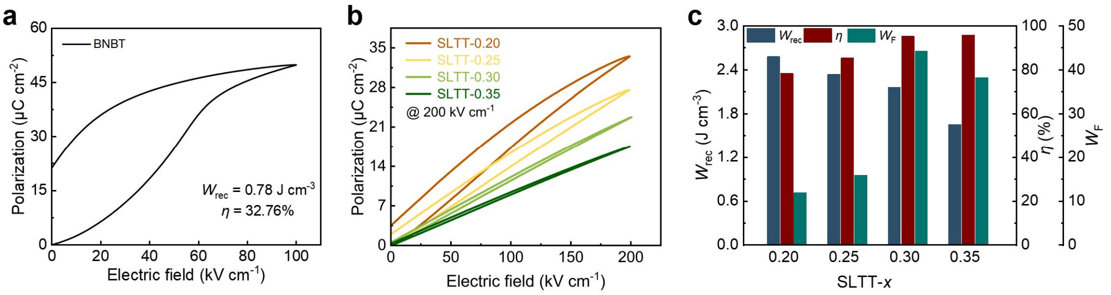

Supplementary Fig. 2 Energy storage performance of SLTT-*x* ceramics under low electric fields. **a** Unipolar *P-E* loop of SLTT-0 ceramic at 100 kV cm^{-1} and 10 Hz. **b** Unipolar *P-E* loops of SLTT-*x* ceramics (x = 0.20, 0.25, 0.30, and 0.35) at 200 kV cm^{-1} and 10 Hz. **c** Comparison of W_{rec}, η, and W_F of SLTT-*x* ceramics (x = 0.20, 0.25, 0.30, and 0.35) at 200 kV cm^{-1}. The total energy density (W_{tot}), W_{rec}, and η can be calculated using the following equations[10,11]:

$$W_{tot} = \int_0^{P_m} E dP \qquad (7)$$

$$W_{rec} = \int_{P_r}^{P_m} E dP \qquad (8)$$

$$\eta = \frac{W_{rec}}{W_{tot}} \times 100\% \qquad (9)$$

Although SLTT-0 ceramic possesses a high P_m, it also has a low E_b and a large P_r, thus resulting in poor performance (W_{rec} = 0.78 J cm^{-3} and η = 32.6%). As x increases, the P_m, polarization switching hysteresis and P_r under 200 kV cm^{-1} demonstrate a decreasing trend. Particularly, SLTT-0.30 and SLTT-0.35 ceramics display extremely thin *P-E* loops, indicating that the increase in S_{config} promotes the transition from RFEs to SPEs. Moreover, the SLTT-0.20 RFE ceramic obtains a high W_{rec} of 2.58 J cm^{-3} but only a moderate η of 78.22%, resulting in a low W_F of 11.83. The SLTT-0.30 SPE ceramic reaches a W_{rec} of 2.33 J cm^{-3} while η increases to 95.11%, and thus the W_F reaches 44.17, which is 273% better than that of SLTT-0.20. The W_{rec} of SLTT-0.35 SPE ceramic decreases to 1.65 J cm^{-3} due to the decrease of P_m to 17.51 μC cm^{-2}, so that the W_F still decreases to 38.16 even though η reaches 95.68%.

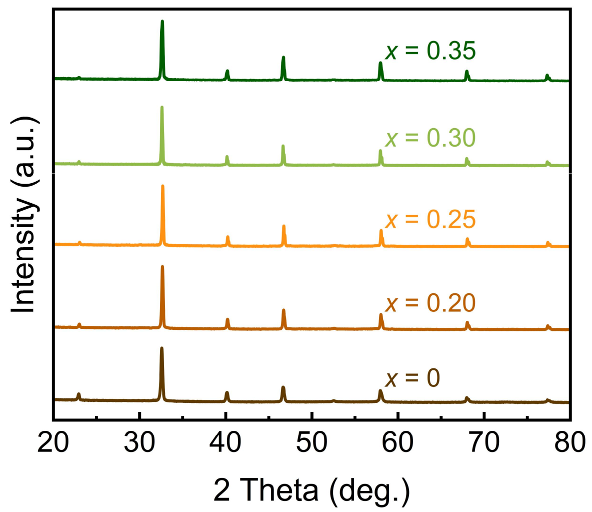

Supplementary Fig. 3 XRD patterns of SLTT-*x* ceramics.

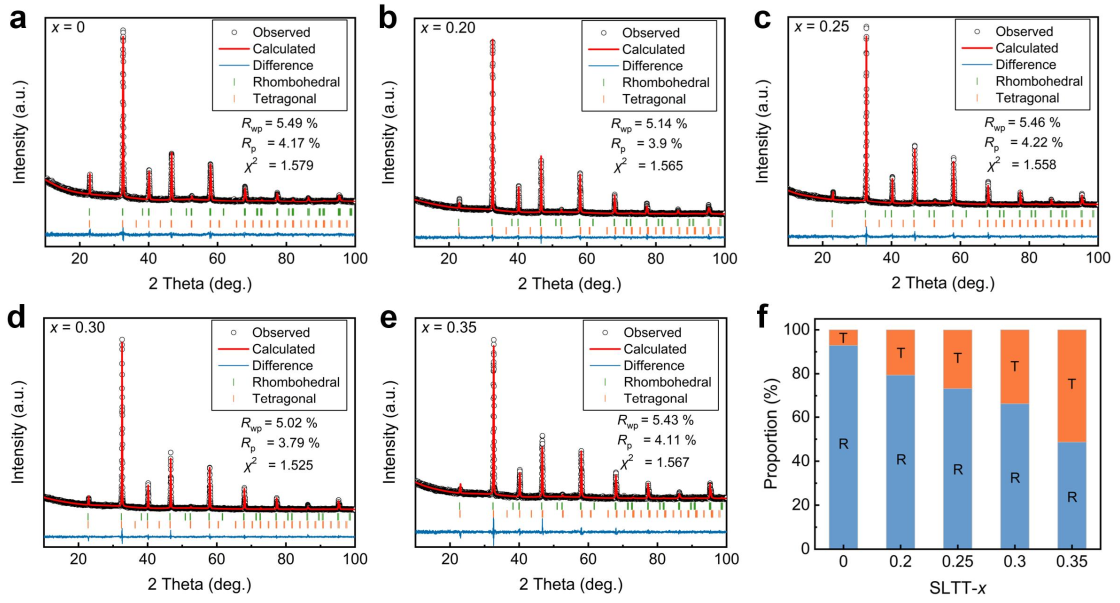

Supplementary Fig. 4 Rietveld refinement results of XRD patterns for SLTT-*x* ceramics. a $x = 0$, **b** $x = 0.20$, **c** $x = 0.25$, **d** $x = 0.30$, **e** $x = 0.35$. **f** Phase proportions of SLTT-*x* ceramics obtained by refinement.

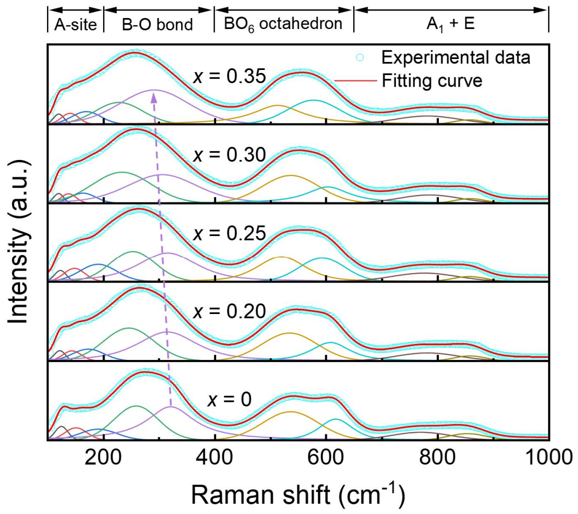

Supplementary Fig. 5 Raman spectra of SLTT-*x* ceramics. Raman spectra (100–1000 cm^{-1}) are used to investigate the local structural variations. Four distinct regions can be observed, which are consistent with the results of previous studies[9,12]. As the SLTT content increases, the peak at ~320 cm^{-1} shifts to lower wavenumbers, indicating increased cation disorder and reduced unit cell polarity[13]. The peak intensity at ~537 cm^{-1} decreases while the peak intensity at ~617 cm^{-1} increases, which is related to the distortion of the BO_6 octahedron[14].

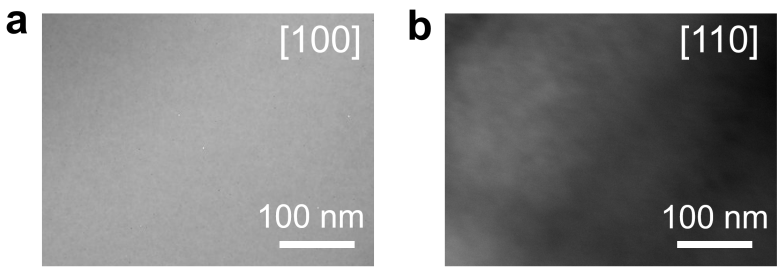

Supplementary Fig. 6 TEM images of SLTT-0.30 ceramic. Low-magnification TEM images along the **a** $[100]_c$ and **b** $[110]_c$ directions.

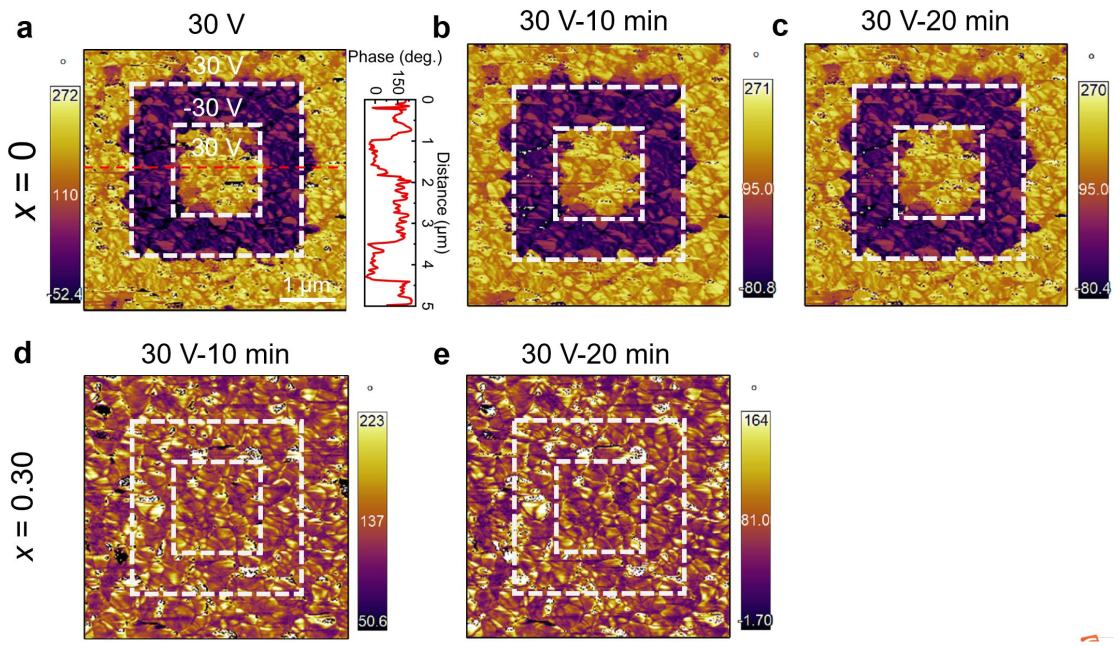

Supplementary Fig. 7 Evolution of domain structure observed by PFM. a Out-of-plane PFM phase images after poling treatment and corresponding piezoresponse phase profiles generated from the line scan for SLTT-0 ceramic. Out-of-plane PFM phase images after relaxation durations, **b**, **c** SLTT-0 and **d**, **e** SLTT-0.30 ceramics. The ferroelectric domain structure and corresponding phase signal are found in the out-of-plane PFM and phase profile of SLTT-0 ceramic. Even after the voltage is unloaded for a period of time, these ferroelectric domains remain in the induced state, which is not conducive to obtaining high energy storage efficiency[9,15]. In contrast, no domain switching is detected in the SLTT-0.30 high-entropy SPE. Such phenomenon may be related to the formation of PNRs. On the one hand, the weak coupling characteristics of PNRs in SPEs delay the formation of large field-induced domains. Therefore, only small-size domains are induced at a voltage of ±30 V, and the limited resolution of PFM makes it difficult to detect the small-size domains induced under this condition. On the other hand, the high dynamic characteristics of PNRs cause the field-induced domains to recover rapidly from the induced state to the initial state after the voltage is unloaded. The PFM images are recorded after voltage unloading, so the domain switching cannot be detected in time[16].

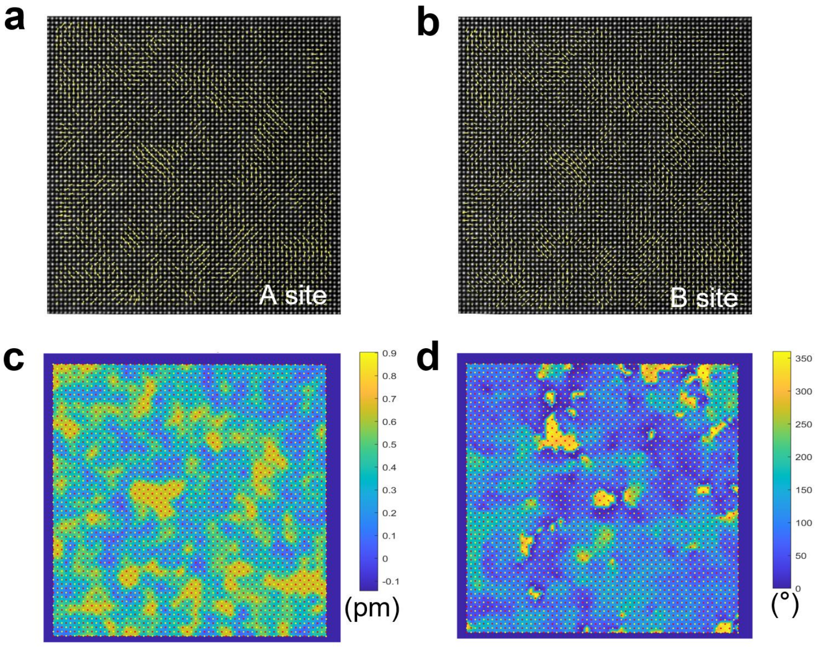

Supplementary Fig. 8 Local polarization configuration of SLTT-0.30 ceramic. Polarization vector mappings of **a** A site and **b** B site atoms along $[100]_c$. **c** Polarization magnitude mapping and **d** polarization angle mapping along $[100]_c$.

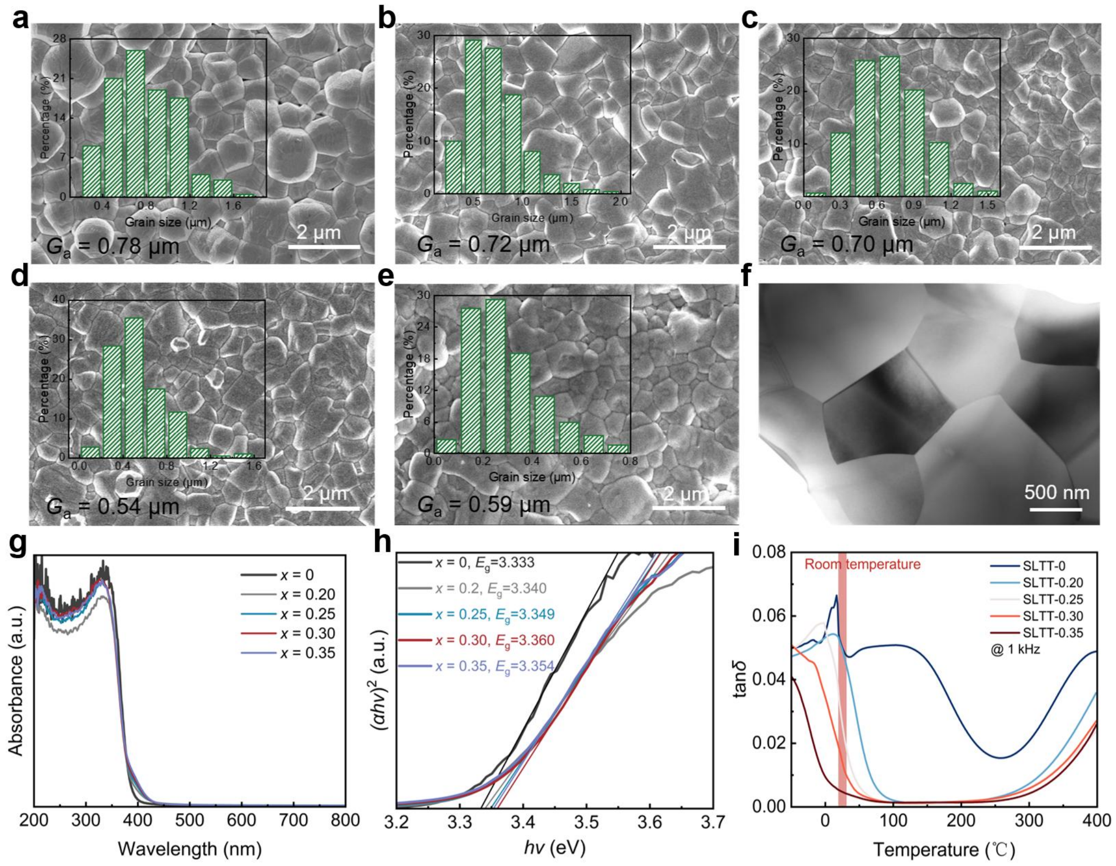

Supplementary Fig. 9 Microstructure, E_g and tanδ of SLTT-x ceramics. Scanning electron microscope (SEM) images and corresponding grain size distributions for **a** SLTT-0, **b** SLTT-0.20, **c** SLTT-0.25, **d** SLTT-0.30, and **e** SLTT-0.35. **f** Grain and grain boundary morphology in TEM image for SLTT-0.30 ceramic. **g** UV-Vis absorption spectra of SLTT-x ceramics. **h** The $(\alpha h\nu)^2$ as a function of $h\nu$ for SLTT-x ceramics. **i** Temperature-dependent tanδ at 1 kHz. SEM images of SLTT-x ceramics reveal a progressively homogeneous microstructure. G_a first decreases and then increases with the increase of x, and the smallest G_a of 0.54 μm is realized in SLTT-0.30 ceramics. TEM image of SLTT-0.30 ceramic shows clear and dense grain boundaries, which can inhibit carrier migration. UV-Vis absorption spectra are further tested, where E_g can be calculated using the Tauc equation[17]:

$$(\alpha h\nu)^2 = A(h\nu - E_g) \qquad (10)$$

where α, $h\nu$, and A are the absorption coefficient, incident photon energy, and proportionality constant, respectively. SLTT-0.30 ceramic possesses the highest E_g of 3.60 eV, which is beneficial to improve the resistivity. Furthermore, SLTT-0.30 ceramic

maintains a low tanδ over a wide temperature range, which provides a guarantee for reducing the probability of thermal failure. Therefore, these factors play a key role in improving E_b[18].

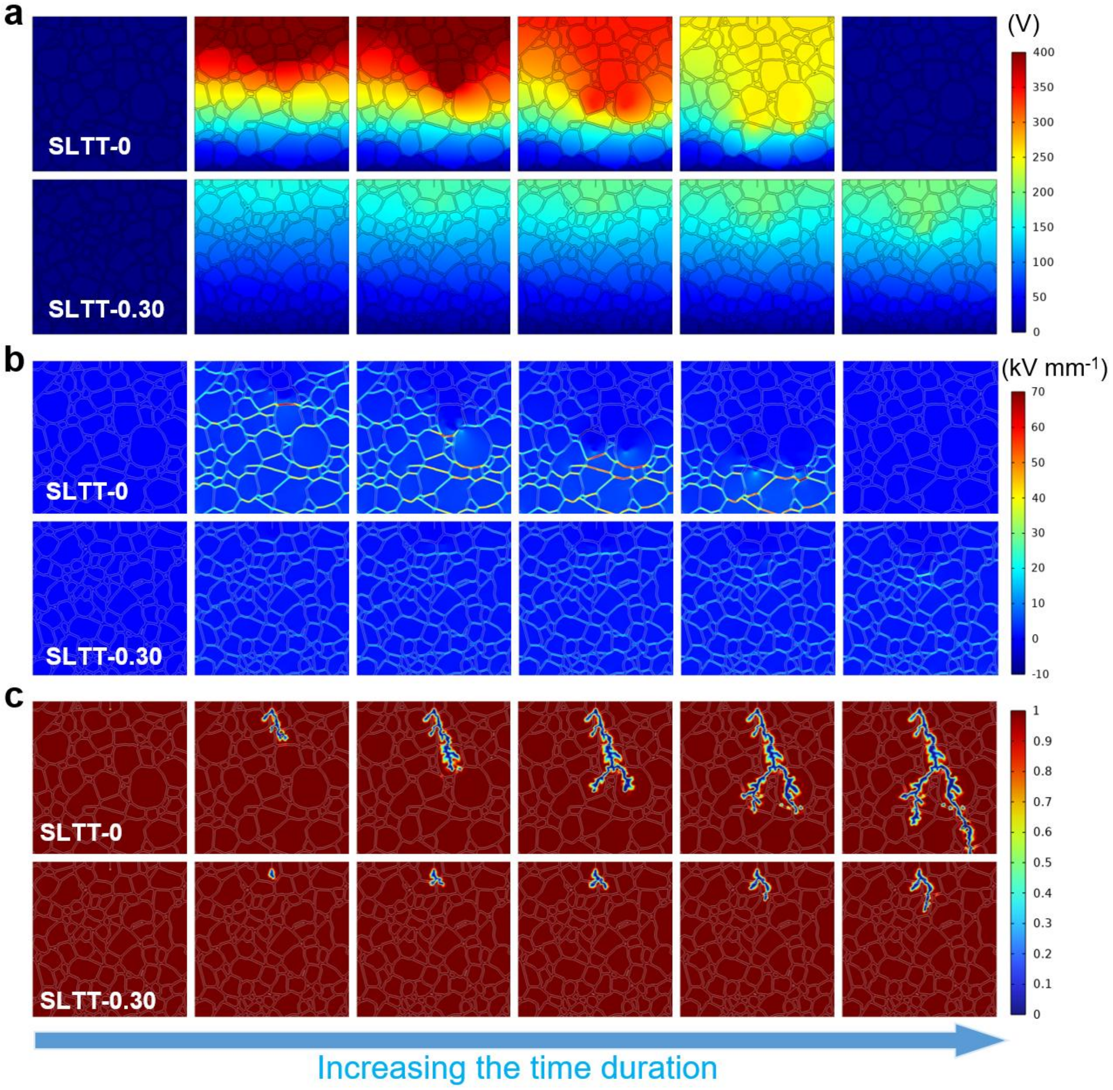

Supplementary Fig. 10 Finite element simulation of electric potential distribution, electric field distribution, and electrical tree evolution. **a** Simulation of electric potential distribution, **b** Simulation of electric field distribution, and **c** Simulation of electrical tree evolution. The SLTT-0 sample exhibits a large local electric field (red area) and a significant electric potential difference under the applied electric field. Meanwhile, the electric tree grows over time, eventually penetrating the entire model. In contrast, the local electric field and potential difference distribution of the SLTT-0.30 sample tends to be uniform, and the transition between grains and grain boundaries is smoother. In addition, the SLTT-0.30 sample shows significantly impeded electric tree propagation, which can be attributed to a more uniform distribution of grains, a higher grain boundary density, and a lower ε_r. These factors lead to energy dissipation during the electric tree propagation process, thus delaying the breakdown[19,20].

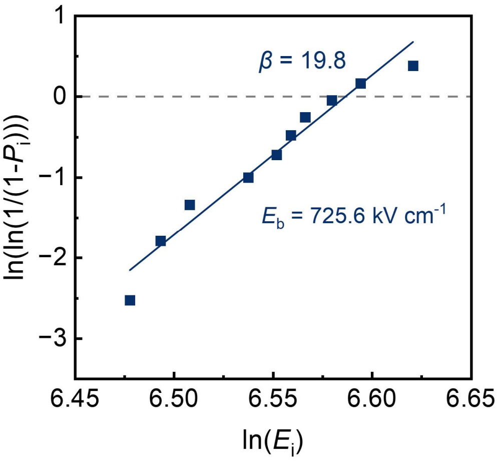

Supplementary Fig. 11 Weibull distribution analysis of SLTT-0.30 ceramics. The Weibull distribution can be calculated using the following equation[21]:

$$P_i = i/(n+1) \qquad (11)$$

$$X_i = \ln{(E_i)} \qquad (12)$$

$$Y_i = \ln{(\ln{(1/(1-P_i))})} \qquad (13)$$

where E_i, i, and n denote the specific breakdown electric field of each sample, the ordinal number of the sample, and the total amount of ceramics, respectively (n is 10 in this work). The slope obtained from the linear fit represents the Weibull modulus (β). The theoretical E_b can be calculated using the intersection of the fitting line with $Y_i = 0$.

Supplementary Table 2 Relevant references for the Fig. 3d and Fig. 3e.

Figure	Reference
Fig. 3d and Fig. 3e	4, 10, 9, 11, 22–96

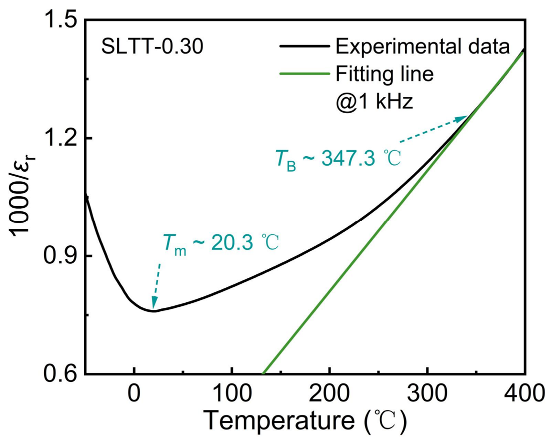

Supplementary Fig. 12 Temperature dependence of 1000/ε_r at 1 kHz for SLTT-0.30 ceramic.

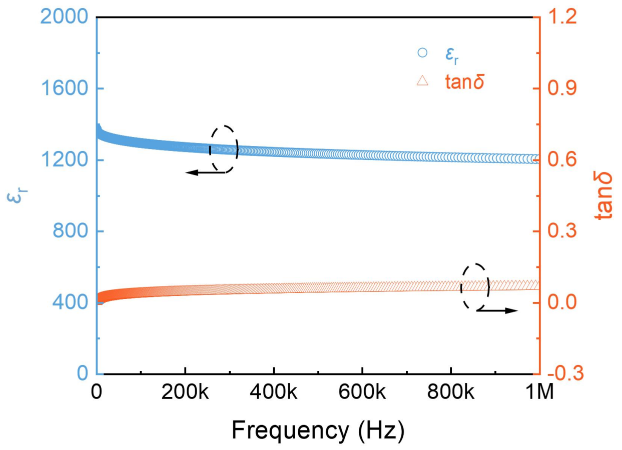

Supplementary Fig. 13 Frequency dependence of ε_r and tanδ for SLTT-0.30 ceramic.

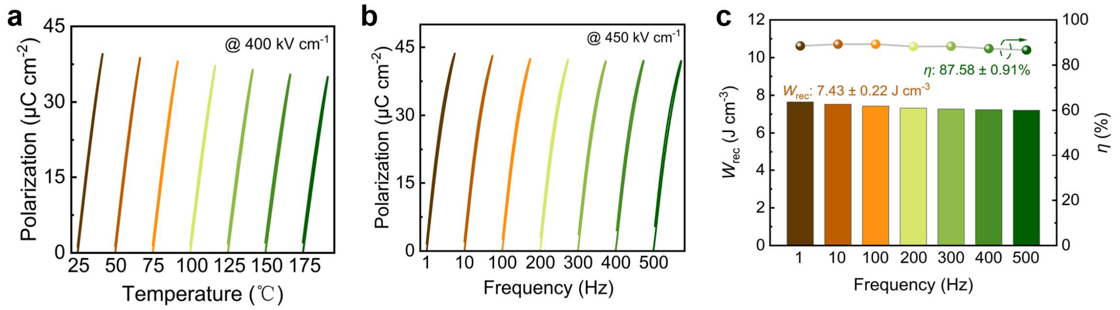

Supplementary Fig. 14 Stability of the SLTT-0.30 ceramic. a Temperature-dependent *P*-*E* loops of SLTT-0.30 ceramic at 400 kV cm^{-1}. **b** Frequency-dependent *P*-*E* loops of SLTT-0.30 ceramic at 450 kV cm^{-1}. **c** Frequency-dependent W_{rec} and η of SLTT-0.30 ceramic at 450 kV cm^{-1}.

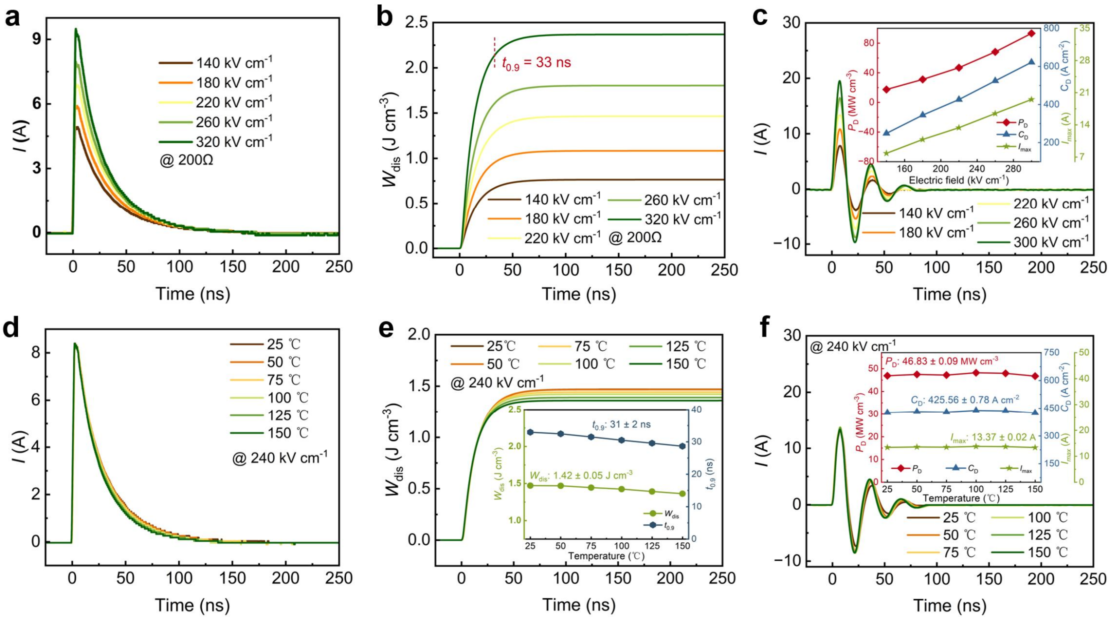

Supplementary Fig. 15 Charge-discharge performance of SLTT-0.30 ceramic. a Overdamped discharge current curves at different electric fields. **b** W_{dis} as a function of time under different electric fields. **c** Underdamped discharge current curves at different electric fields. The inset shows I_{max}, C_D, and P_D as a function of electric field. **d** Temperature-dependent overdamped discharge current curves at 240 kV cm^{-1}. **e** W_{dis} as a function of time under different temperatures. The inset shows W_{dis} and $t_{0.9}$ as a function of temperature. **f** Temperature-dependent underdamped discharge current curves at 240 kV cm^{-1}. The inset shows I_{max}, C_D, and P_D as a function of temperature.

The discharge energy density W_{dis}, current density C_D and power density P_D can be calculated using the following equations[22]:

$$W_{dis} = R\frac{\int I^2(t)dt}{V} \qquad (14)$$

$$C_D = \frac{I_{max}}{S} \qquad (15)$$

$$P_D = \frac{E \times I_{max}}{2S} \qquad (16)$$

where R, V, and S are the load resistor (200 Ω), volume, and electrode area, respectively.

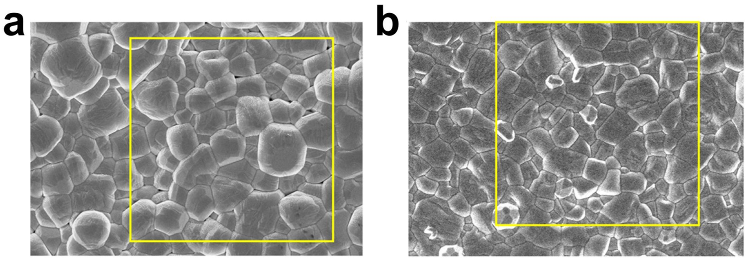

Supplementary Fig. 16 SEM images for finite element simulation. a SLTT-0, **b** SLTT-0.30. The yellow boxed areas indicate selected regions.

References

1. Cai, Z. et al. Dielectric response and breakdown behavior of polymer-ceramic nanocomposites: the effect of nanoparticle distribution. *Compos. Sci. Technol.* **145**, 105–113 (2017).
2. Chen, L. et al. Large energy capacitive high-entropy lead-free ferroelectrics. *Nano-Micro Lett.* **15**, 65 (2023).
3. Johnson, K. Variation of dielectric constant with voltage in ferroelectrics and its application to parametric devices. *J. Appl. Phys.* **33**, 2826–2831 (1962).
4. Dong, X. et al. (1-x)[0.90NN-0.10Bi($Mg_{2/3}Nb_{1/3}$)O_3]-x($Bi_{0.5}Na_{0.5}$)$_{0.7}Sr_{0.3}TiO_3$ ceramics with core–shell structures: a pathway for simultaneously achieving high polarization and breakdown strength. *Nano Energy* **101**, 107577 (2022).
5. Padurariu, L. et al. Field-dependent permittivity in nanostructured $BaTiO_3$ ceramics: modeling and experimental verification. *Phys. Rev. B* **85**, 224111 (2012).
6. Yang, B. et al. Engineering relaxors by entropy for high energy storage performance. *Nat. Energy* **8**, 956–964 (2023).
7. Yang, B. et al. High-entropy enhanced capacitive energy storage. *Nat. Mater.* **21**, 1074–1080 (2022).
8. Lu, Z. et al. Superior energy density through tailored dopant strategies in multilayer ceramic capacitors. *Energy Environ. Sci.* **13**, 2938–2948 (2020).
9. Zhu, X. et al. Ultrahigh energy storage density in ($Bi_{0.5}Na_{0.5}$)$_{0.65}Sr_{0.35}TiO_3$-based lead-free relaxor ceramics with excellent temperature stability. *Nano Energy* **98**, 107276 (2022).
10. Zhang, M. et al. Significant increase in comprehensive energy storage performance of potassium sodium niobate-based ceramics via synergistic optimization strategy. *Energy Storage Mater.* **45**, 861–868 (2022).

11. Xie, A. et al. Supercritical relaxor nanograined ferroelectrics for ultrahigh-energy-storage capacitors. *Adv. Mater.* **34**, 2204356 (2022).
12. Che, Z. et al. Phase structure and defect engineering in $(Bi_{0.5}Na_{0.5})TiO_3$-based relaxor antiferroelectrics toward excellent energy storage performance. *Nano Energy* **100**, 107484 (2022).
13. Yan, F. et al. Superior energy storage properties and excellent stability achieved in environment-friendly ferroelectrics via composition design strategy. *Nano Energy* **75**, 105012 (2020).
14. Yan, F. et al. Optimization of polarization and electric field of bismuth ferrite-based ceramics for capacitor applications. *Chem. Eng. J.* **417**, 127945 (2021).
15. Wang, Z. et al. $(Bi_{0.5}Na_{0.5})TiO_3$-based relaxor ferroelectrics with medium permittivity featuring enhanced energy-storage density and excellent thermal stability. *Chem. Eng. J.* **427**, 131989 (2022).
16. Liu, L. et al. Multi-scale collaborative optimization of $SrTiO_3$-based energy storage ceramics with high performance and excellent stability. *Nano Energy* **109**, 108275 (2023).
17. Cao, W. et al. Phase and band structure engineering via linear additive in NBT-ST for excellent energy storage performance with superior thermal stability. *ACS Appl. Mater. Interfaces* **14**, 54051–54062 (2022).
18. Liu, J. et al. Giant comprehensive capacitive energy storage in lead-free quasi-linear relaxor ferroelectrics via local heterogeneous polarization configuration. *J. Mater. Chem. A* **11**, 15931–15942 (2023).
19. Li, X. et al. Simultaneous enhancement of energy storage and hardness performances in $(Na_{0.5}Bi_{0.5})_{0.7}Sr_{0.3}TiO_3$-based relaxor ferroelectrics via multiscale regulation. *ACS Appl. Mater. Interfaces* **14**, 42245–42257 (2022).
20. Wang, W. et al. Enhancing energy storage performance in $Na_{0.5}Bi_{0.5}TiO_3$-based lead-free relaxor ferroelectric ceramics along a stepwise optimization route. *J. Mater. Chem. A* **11**, 2641–2651 (2023).
21. Chen, L. et al. Near-zero energy consumption capacitors by controlling inhomogeneous polarization configuration. *Adv. Mater.* **36**, 2313285 (2024).
22. Chai, Q. et al. Superior energy storage properties and optical transparency in $K_{0.5}Na_{0.5}NbO_3$-based dielectric ceramics via multiple synergistic strategies. *Small* **19**, 2207464 (2023).
23. Hu, Q. et al. Achieve ultrahigh energy storage performance in $BaTiO_3$-Bi

$(Mg_{1/2}Ti_{1/2})O_3$ relaxor ferroelectric ceramics via nano-scale polarization mismatch and reconstruction. *Nano Energy* **67**, 104264 (2020).

24. Huang, W. et al. Superior energy storage performances achieved in (Ba, Sr)TiO_3-based bulk ceramics through composition design and core-shell structure engineering. *Chem. Eng. J.* **444**, 135523 (2022).
25. Chen, L. et al. Excellent energy storage and mechanical performance in hetero-structure $BaTiO_3$-based relaxors. *Chem. Eng. J.* **452**, 139222 (2023).
26. Sun, Z. et al. Superior capacitive energy-storage performance in Pb-free relaxors with a simple chemical composition. *J. Am. Chem. Soc.* **145**, 6194–6202 (2023).
27. Zhao, J. et al. Delayed polarization saturation induced superior energy storage capability of $BiFeO_3$-based ceramics via introduction of non-isovalent ions. *Small* **19**, 2206840 (2023).
28. Yan, F. et al. Composition and structure optimized $BiFeO_3$-$SrTiO_3$ lead-free ceramics with ultrahigh energy storage performance. *Small* **18**, 2106515 (2022).
29. Qi, H. et al. Superior energy-storage capacitors with simultaneously giant energy density and efficiency using nanodomain engineered $BiFeO_3$-$BaTiO_3$-$NaNbO_3$ lead-free bulk ferroelectrics. *Adv. Energy Mater.* **10**, 1903338 (2019).
30. Li, D. et al. Improved energy storage properties achieved in (K, Na)NbO_3-based relaxor ferroelectric ceramics via a combinatorial optimization strategy. *Adv. Funct. Mater.* **32**, 2111776 (2021).
31. Chen, L. et al. Chen, L. et al. Giant energy-storage density with ultrahigh efficiency in lead-free relaxors via high-entropy design. *Nat. Commun.* **13**, 3089 (2022).
32. Qi, H. et al. Linear-like lead-free relaxor antiferroelectric $(Bi_{0.5}Na_{0.5})TiO_3$-$NaNbO_3$ with giant energy-storage density/efficiency and super stability against temperature and frequency. *J. Mater. Chem. A* **7**, 3971–3978 (2019).
33. Zhang, Y. et al. Superior energy-storage properties in $Bi_{0.5}Na_{0.5}TiO_3$-based lead-Free ceramics via simultaneously manipulating multiscale structure and field-induced structure transition. *ACS Appl. Mater. Interfaces* **14**, 40043–40051 (2022).
34. Ji, H. et al. Ultrahigh energy density in short-range tilted NBT-based lead-free multilayer ceramic capacitors by nanodomain percolation. *Energy Storage Mater.* **38**, 113–120 (2021).
35. Yan, F. et al. Excellent energy storage properties and superior stability achieved in lead-free ceramics via a spatial sandwich structure design strategy. *J. Mater. Chem.*

A **9**, 15827–15835 (2021).

36. Yan, F. et al. Boosting energy storage performance of lead-free ceramics via layered structure optimization strategy. *Small* **18**, 2202575 (2022).
37. Che, Z. et al. Phase structure and defect engineering in $(Bi_{0.5}Na_{0.5})TiO_3$-based relaxor antiferroelectrics toward excellent energy storage performance. *Nano Energy* **100**, 107484 (2022).
38. Wang, W. et al. Enhanced energy storage properties in lead-free $(Na_{0.5}Bi_{0.5})_{0.7}Sr_{0.3}TiO_3$-based relaxor ferroelectric ceramics through a cooperative optimization strategy. *ACS Appl. Mater. Interfaces* **15**, 6990–7001 (2023).
39. Wang, W. et al. Enhancing energy storage performance in $Na_{0.5}Bi_{0.5}TiO_3$-based lead-free relaxor ferroelectric ceramics along a stepwise optimization route. *J. Mater. Chem. A* **11**, 2641–2651 (2023).
40. Cao, W. et al. Interfacial polarization restriction for ultrahigh energy-storage density in lead-Free ceramics. *Adv. Funct. Mater.* **33**, 2301027 (2023).
41. Liu, H. et al. Chemical design of Pb-free relaxors for giant capacitive energy storage. *J. Am. Chem. Soc.* **145**, 11764–11772 (2023).
42. Tang, L. et al. Giant energy storage density with antiferroelectric-like properties in BNT-based ceramics via phase structure engineering. *Small* **19**, 2302346 (2023).
43. He, B. et al. Excellent energy storage performance of $(Sc_{0.5}Ta_{0.5})^{4+}$ modified $(Bi_{0.5}Na_{0.5})TiO_3$-based ceramics modulated by the evolution of polar phases. *J. Mater. Chem. A* **11**, 14169–14179 (2023).
44. Wang, Q. et al. $Bi_{0.5}Na_{0.5}TiO_3$-based relaxor-ferroelectric ceramics for low-electric-field dielectric energy storage via bidirectional optimization strategy. *Chem. Eng. J.* **452**, 139422 (2023).
45. Dai, Z. et al. Improved energy storage density and efficiency of $(1–x)Ba_{0.85}Ca_{0.15}Zr_{0.1}Ti_{0.9}O_3$-$x$$BiMg_{2/3}Nb_{1/3}O_3$ lead-free ceramics. *Chem. Eng. J.* **410**, 128341 (2020).
46. Shen, Y. et al. Constructing novel binary $Bi_{0.5}Na_{0.5}TiO_3$-based composite ceramics for excellent energy storage performances via defect engineering. *Chem. Eng. J.* **439**, 135762 (2022).
47. Li, X. et al. Realizing excellent energy storage properties in $Na_{0.5}Bi_{0.5}TiO_3$-based lead-free relaxor ferroelectrics. *J. Eur. Ceram. Soc.* **42**, 2221–2229 (2022).
48. Wu, L. et al. Enhanced energy-storage performance in BNT-based lead-free dielectric ceramics via introducing $SrTi_{0.875}Nb_{0.1}O_3$. *J. Materiomics* **8**, 537–544

(2022).

49. Li, T. et al. Energy storage performance of $Na_{0.5}Bi_{0.5}TiO_3$-$SrTiO_3$ lead-free relaxors modified by $AgNb_{0.85}Ta_{0.15}O_3$. *Chem. Eng. J.* **406**, 127151 (2021).
50. Li, C. et al. Superior energy storage performance in $(Bi_{0.5}Na_{0.5})TiO_3$-based lead-free relaxor ferroelectrics for dielectric capacitor application via multiscale optimization design. *J. Mater. Chem. A*, **10**, 9535–9546 (2022).
51. Kang, R. et al. Enhanced energy storage performance in $Sr_{0.7}La_{0.2}Zr_{0.15}Ti_{0.85}O_3$-modified $Bi_{0.5}Na_{0.5}TiO_3$ ceramics via constructing local phase coexistence. *Chem. Eng. J.* **446**, 137105 (2022).
52. Guo, B. et al. Energy storage performance of $Na_{0.5}Bi_{0.5}TiO_3$ based lead-free ferroelectric ceramics prepared via non-uniform phase structure modification and rolling process. *Chem. Eng. J.* **420**, 130475 (2021).
53. Ye, H. et al. Significantly improvement of comprehensive energy storage performances with lead-free relaxor derroelectric ceramics for high-temperature capacitors applications. *Acta Mater.* **203**, 116484 (2021).
54. Ding, Y. et al. Simultaneously achieving high energy-storage efficiency and density in Bi-modified $SrTiO_3$-based relaxor ferroelectrics by ion selective engineering. *Compos. B. Eng.* **230**, 109493 (2022).
55. Yang, Q. et al. Excellent energy storage performance of $K_{0.5}Bi_{0.5}TiO_3$-based ferroelectric ceramics under low electric field. *Chem. Eng. J.* **414**, 128769 (2021).
56. Li, D. et al. Enhanced energy storage properties achieved in $Na_{0.5}Bi_{0.5}TiO_3$-based ceramics via composition design and domain engineering. *Chem. Eng. J.* **419**, 129601 (2021).
57. Huang, J. et al. Expanded linear polarization response and excellent energy-storage properties in $(Bi_{0.5}Na_{0.5})TiO_3$-$KNbO_3$ relaxor antiferroelectrics with medium permittivity. *Chem. Eng. J.* **398**, 125639 (2020).
58. Li, X. et al. Simultaneous enhancement of energy storage and hardness performances in $(Na_{0.5}Bi_{0.5})_{0.7}Sr_{0.3}TiO_3$-based relaxor ferroelectrics via multiscale regulation. *ACS Appl. Mater. Interfaces* **14**, 42245–42257 (2022).
59. Xie, A. et al. $NaNbO_3$-$CaTiO_3$ lead-free relaxor antiferroelectric ceramics featuring giant energy density, high energy efficiency and power density. *Chem. Eng. J.* **429**, 132534 (2022).
60. Xie, A. et al. $NaNbO_3$-$(Bi_{0.5}Li_{0.5})TiO_3$ lead-free relaxor ferroelectric capacitors with superior energy-storage performances via multiple synergistic design. *Adv.*

Energy Mater. **11**, 2101378 (2021).

61. Chen, H. et al. Excellent energy storage properties and stability of $NaNbO_3$-$Bi(Mg_{0.5}Ta_{0.5})O_3$ ceramics by introducing $(Bi_{0.5}Na_{0.5})_{0.7}Sr_{0.3}TiO_3$. *J. Mater. Chem. A* **9**, 4789–4799 (2021).
62. Zhou, M. et al. Superior energy storage properties and excellent stability of novel $NaNbO_3$-based lead-free ceramics with A-site vacancy obtained via a Bi_2O_3 substitution strategy. *J. Mater. Chem. A* **6**, 17896–17904 (2018).
63. Yang, W. et al. Superior energy storage properties in $NaNbO_3$-based ceramics via synergistically optimizing domain and band structures. *J. Mater. Chem. A* **10**, 11613–11624 (2022).
64. Xu, Z. et al. Simultaneously achieving large energy density and high efficiency in $NaNbO_3$-$(Sr,Bi)TiO_3$-$Bi(Mg,Zr)O_3$ relaxor ferroelectric ceramics for dielectric capacitor applications. *J. Mater. Chem. A* **10**, 13907–13916 (2022).
65. Pang, F. et al. $Bi(Mg_{0.5}Sn_{0.5})O_3$-doped $NaNbO_3$ lead-free ceramics achieve excellent energy-storage and charge/discharge performances. *ACS Sustain. Chem. Eng.* **9**, 4863–4871 (2021).
66. Dong, X. et al. High energy storage density and power density achieved simultaneously in $NaNbO_3$-based lead-free ceramics via antiferroelectricity enhancement. *J. Materiomics* **7**, 629–639 (2021).
67. Qiao, Z. et al. Excellent energy storage properties in $NaNbO_3$-based lead-free ceramics by modulating antiferrodistortive of P phase. *J. Alloys Compd.* **898**, 162934 (2022).
68. Chen, Z. et al. Phase engineering in $NaNbO_3$ antiferroelectrics for high energy storage density. *J. Materiomics* **8**, 753–762 (2022).
69. Liu, G. et al. Energy storage properties of $NaNbO_3$-based lead-free superparaelectrics with large antiferrodistortion. *Microstructures*, **3**, 2023009 (2023).
70. Pang, F. et al. Ultrahigh energy storage characteristics of sodium niobate-based ceramics by introducing a local random field. *ACS Sustain. Chem. Eng.* **8**, 14985–14995 (2020).
71. Chen, H. et al. Adjusting the energy-storage characteristics of $0.95NaNbO_3$-$0.05Bi(Mg_{0.5}Sn_{0.5})O_3$ ceramics by doping linear perovskite materials. *ACS Appl. Mater. Interfaces*, **14**, 25609–25619 (2022).
72. Ma, J. et al. Ultrahigh energy storage density and high efficiency in lead-free

$(Bi_{0.9}Na_{0.1})(Fe_{0.8}Ti_{0.2})O_3$-modified $NaNbO_3$ ceramics via stabilizing the antiferroelectric phase and enhancing relaxor behavior. *ACS Appl. Mater. Interfaces* **14**, 19704–19713 (2022).

73. Zhi, L. et al. Mechanism of enhanced energy storage density in $AgNbO_3$-based lead-free antiferroelectrics. *Nano Energy* **79**, 105423 (2021).
74. Li, S. et al. Giant energy density and high efficiency achieved in silver niobate-based lead-free antiferroelectric ceramic capacitors via domain engineering. *Energy Storage Mater.* **34**, 417–426 (2021).
75. Luo, N. et al. Constructing phase boundary in $AgNbO_3$ antiferroelectrics: pathway simultaneously achieving high energy density and efficiency. *Nat. Commun.* **11**, 4824 (2020).
76. Fan, X. et al. Synergic enhancement of energy storage density and efficiency in MnO_2-doped $AgNbO_3$@SiO_2 ceramics via A/B-Site substitutions. *ACS Appl. Mater. Interfaces* **14**, 7052–7062 (2022).
77. Liu, L. et al. Multi-scale collaborative optimization of $SrTiO_3$-based energy storage ceramics with high performance and excellent stability. *Nano Energy* **109**, 108275 (2023).
78. Zhao, P. et al. Structure, dielectric and relaxor properties of $Sr_{0.7}Bi_{0.2}TiO_3$-$K_{0.5}Bi_{0.5}TiO_3$ lead-free ceramics for energy storage applications. *J. Materiomics*, 7, 195–207 (2021).
79. Zuo, C. et al. Excellent energy storage and hardness performance of $Sr_{0.7}Bi_{0.2}TiO_3$ ceramics fabricated by solution combustion-synthesized nanopowders. *Chem. Eng. J.* **442**, 136330 (2022).
80. Li, C. et al. Superior energy storage capability and stability in lead-free relaxors for dielectric capacitors utilizing nanoscale polarization heterogeneous regions. *Small* **19**, 2206662 (2023).
81. Chen, L. et al. Local diverse polarization optimized comprehensive energy-storage performance in lead-free superparaelectrics. *Adv. Mater.* **34**, 2205787 (2022).
82. Chen, L. et al. Large energy capacitive high-entropy lead-free ferroelectrics. *Nano-Micro Lett.* **15**, 65 (2023).
83. Guo, J. et al. Multi-symmetry high-entropy relaxor ferroelectric with giant capacitive energy storage. *Nano Energy* **112**, 108458 (2023).
84. Yan, B. et al. Design and preparation of lead-free $(Bi_{0.4}Na_{0.2}K_{0.2}Ba_{0.2})TiO_3$-$Sr(Mg_{1/3}Nb_{2/3})O_3$ high-entropy relaxor ceramics for dielectric energy storage.

Chem. Eng. J. **453**, 139921 (2023).

85. Wang, W. et al. Enhanced energy storage and fast charge-discharge capability in $Ca_{0.5}Sr_{0.5}TiO_3$-based linear dielectric ceramic. *J. Alloys and Compd.* **817**, 152695 (2020)
86. Ye, J. et al. Excellent comprehensive energy storage properties of novel lead-free $NaNbO_3$-based ceramics for dielectric capacitor applications. *J. Mater. Chem. C.* **7**, 5639–5645 (2019).
87. Yang, D. et al. Composition design of BNBT-ST relaxor ferroelectric ceramics in superparaelectric state with ultrahigh energy density. *Ceram. Int.* **49**, 27750–27757 (2023).
88. Ye, W. et al. Enhanced energy-storage properties in Zr^{4+}-modified $(Bi_{0.4}Ba_{0.2}K_{0.2}Na_{0.2})TiO_3$ high-entropy ceramics. *J. Am. Ceram. Soc.* **106**, 6858–6867 (2023).
89. Pu, Y. et al. Enhancing the energy storage properties of $Ca_{0.5}Sr_{0.5}TiO_3$-based lead-free linear dielectric ceramics with excellent stability through regulating grain boundary defects. *J. Mater. Chem. C.* **7**, 14384–14393 (2019).
90. Luo, B. et al. Enhanced energy-storage density and high efficiency of lead-free $CaTiO_3$–$BiScO_3$ linear dielectric ceramics. *ACS Appl. Mater. Interfaces* **9**, 19963–19972 (2017).
91. Liu, J. et al. Giant comprehensive capacitive energy storage in lead-free quasi-linear relaxor ferroelectrics via local heterogeneous polarization configuration. *J. Mater. Chem. A* **11**, 15931–15942 (2023).
92. Li, J. et al.. Enhanced energy storage performance under low electric field in Sm^{3+} doped $AgNbO_3$ ceramics. *J. Materiomics* **8**, 266–273 (2022).
93. Fan, J. et al. Ultrahigh energy storage performance of a $0.75Bi_{0.47}Na_{0.47}Ba_{0.06}TiO_3$-$0.25CaTi_{0.8}Sn_{0.2}O_3$ ceramic under moderate electric fields. *Inorg. Chem. Front.* **10**, 5475–5487 (2023).
94. Huang, J. et al. Greatly enhanced energy storage and discharge properties of $AgNbO_3$ ceramics with a stable antiferroelectric phase and high breakdown strength using hydrothermally synthesized powders. *J. Mater. Chem. A* **10**, 16337–16350 (2022).
95. Wu, S. et al. Superb energy storage capability for $NaNbO_3$-based ceramics featuring labyrinthine submicro-domains with clustered lattice distortions. *Small* **19**, 2303915 (2023).

96. Li, D. et al. A high-temperature performing and near-zero energy loss lead-free ceramic capacitor. *Energy Environ. Sci.* **16**, 4511–4521 (2023).
